# Shedding light on PSII components in the dark

**DOI:** 10.1093/plcell/koae254

**Published:** 2024-09-18

**Authors:** Nora Flynn

**Affiliations:** Assistant Features Editor, The Plant Cell, American Society of Plant Biologists; Department of Botany and Plant Sciences, University of California Riverside, CA 92507, USA

Plastids are multifaceted organelles that perform diverse cellular functions in plant cells, including energy production by photosynthetic chloroplasts. The light-dependent reactions of photosynthesis take place in an inner chloroplast membrane network, the thylakoid, which is composed of disk-like, stacked grana membranes interconnected by unstacked stroma lamellae (see Fig. 1A). Embedded in the thylakoid membrane, two photosystem complexes harvest light energy, with PSII catalyzing water splitting in the initial step of the photosynthetic electron transport chain.

**Figure 1.**
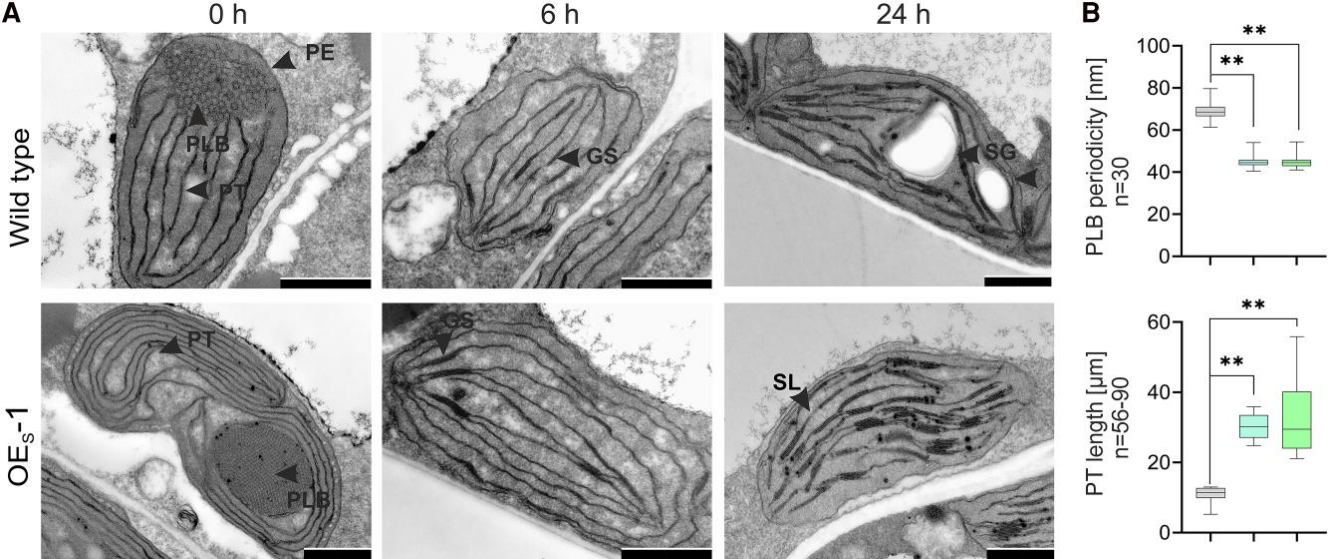
Prothylakoid “snailing” in OE_S_ etioplasts. **A)** Representative transmission electron microscopy images from OE_S_ and wild-type dark-grown etioplasts (0 h) or developing chloroplasts after 6 or 24 h of light exposure. PLB, prolamellar body; PT, prothylakoid; PE, plastid envelope; GS, grana stack; SG, starch grain; SL, stroma lamella. Scar bar = 1 *µ*m. **B)** Quantification of prothylakoid and prolamellar body characteristics in etioplasts. Adapted from Li et al. (2024), Figure 5.

The assembly and function of many protein subunits within the PSII complex remain an area of active research (Komenda et al. 2024). Decoding the functions of the PSII cytochrome *b*_559_ has proven especially challenging. Cytochrome *b*_559_ has structural roles in PSII and is a heterodimer that is made of 2 plastid-encoded proteins, PsbE and PsbF (Cramer and Zakharov 2022). Due to its effect on PSII structure, disturbance of cytochrome *b*_559_ leads to a reduction of PSII as a whole, which in turn causes light-sensitive phenotypes (Swiatek et al. 2003). The loss of PSII activity makes it difficult to interpret the specific activities of cytochrome *b*_559_ outside of structural support. **Bingqi Li, Tegan Armarego-Marriott, and colleagues** (Li et al. 2024) explore the significance of cytochrome *b*_559_ by instead unraveling the role of one of its components in the dark, allowing them to circumvent the widescale phenotypic effects when PSII function is altered. By focusing on etioplasts, nonphotosynthetic, dark-grown plastids that transform into chloroplasts with light, the authors revealed a possible role of PSII components in the regulation of lipid biosynthesis during the early stages of thylakoid biogenesis.

Loss of cytochrome *b*_559_ does not support autotrophic growth (Swiatek et al. 2003), so the authors investigated cytochrome *b*_559_ by overexpressing the plastid-encoded operon containing *psbE* and *psbF* in *Nicotiana tabacum*. To see a progressive effect on plant phenotypes, the authors tested three different constructs, each with different levels of overexpression, referred to as weak overexpressing (OE_W_), medium overexpressing (OE_M_), or strong overexpressing (OE_S_) lines. After transforming each construct into plants, an increasingly severe phenotype was observed when plants were grown in light. OE_S_ lines demonstrated the most severe phenotype, with pale leaves, slow growth, delayed greening, and necrosis, suggesting increased photooxidative damage. However, these phenotypes were less apparent in low-light conditions and disappeared in darkness, the latter opening an avenue to explore the role of cytochrome *b*_559_ outside of photosynthesis.

PsbE and PsbF have unknown roles in etioplasts but are known to be present, even though PSII is not yet assembled (Plöscher et al. 2009). As expected, OE_S_ etioplasts lacked PSII complexes but displayed a high level of PsbE, estimated to be tenfold more than wild-type levels. This was accompanied by a moderate increase in cytochrome *b*_559_, suggesting additional assembly factors may influence cytochrome *b*_559_ appearance. Alongside the elevated level of PsbE, OE_S_ etioplasts had striking ultrastructural differences, indicating that components of the PSII complex may influence early membrane development (see Fig. 1). OE_S_ etioplasts had extensive layers of thylakoid precursors, called prothylakoids, that created a prothylakoid “snailing” phenotype. The prothylakoids wrapped around the prolamellar body, a membrane lattice with a similarly unusual, condensed organization in OE_S_ etioplasts. However, these pronounced membrane phenotypes disappeared upon exposure to light, where OE_S_ chloroplasts more closely resembled wild type (see Fig. 1A).

How can OE_S_ etioplasts support such a drastic proliferation of membranes? Using an ultra performance liquid chromatography-mass spectrometry (UPLC-MS)–based lipidomic approach, the authors demonstrated an increased accumulation of specific lipid classes in OE_S_ lines compared to wild type. This suggests a model where the availability of membrane proteins, like PsbE, could influence plastid lipid synthesis, regulating the rate of prothylakoid biogenesis. Further, the effect of PsbE overaccumulation on etioplast ultrastructure reveals the potential for a novel role of cytochrome *b*_559_ components outside of photosynthesis. The precise mechanism of how membrane protein provision informs prothylakoid biogenesis requires further study but may provide insights into broader regulatory pathways during the biosynthesis of diverse biological membranes.

## Data Availability

As an in-brief article, there is no accompanying data to declare.

## Dive Curated Terms

The following phenotypic, genotypic, and functional terms are of significance to the work described in this paper:

cytochrome CHEBI: CHEBI:4056
